# A Systematic Review of Community-Acquired Pneumonia in Indian Adults

**DOI:** 10.7759/cureus.63976

**Published:** 2024-07-06

**Authors:** Vikram B Vikhe, Ahsan A Faruqi, Rahul S Patil, Avani Reddy, Devansh Khandol

**Affiliations:** 1 General Medicine, Dr. D. Y. Patil Medical College, Hospital & Research Centre, Dr. D. Y. Patil Vidyapeeth, Pune (Deemed to be University), Pune, IND

**Keywords:** antibiotic resistance (abr), a systematic review, community-acquired pneumonia (cap), empirical antibiotic therapy, india, indian adults, klebsiella pneumoniae (kp), pneumonia, streptococcus pneumoniae

## Abstract

This systematic review aimed to consolidate findings on the etiology of community-acquired pneumonia (CAP) among Indian adults. We adhered to the Preferred Reporting Items for Systematic reviews and Meta-Analyses (PRISMA) Guidelines 2020 and conducted a comprehensive search across databases including PubMed, Scopus-Elsevier, and hand-searched reference lists using key terms such as “Community-Acquired Pneumonia,” “CAP,” “Indian,” and “adults.” Articles published between January 2010 and January 2024 were included, with exclusions for studies involving pediatric populations, non-Indian patients, or those published before 2010. From an initial pool of 344 articles, duplicates were removed and titles and abstracts were screened, resulting in nine studies meeting the inclusion criteria. The analysis of pooled data comprising 1,643 Indian adult participants revealed the following pathogen distribution: *Streptococcus pneumoniae* was the most common organism, accounting for 33% of the cases. This was followed by *Klebsiella pneumoniae* at 23%, *Staphylococcus aureus* at 10%, *Mycoplasma pneumoniae* and *Legionella pneumophila* each at 7%, and *Chlamydia pneumoniae*, *Haemophilus influenzae*, and *Pseudomonas aeruginosa* each at 4%. Notably, the review highlights a rising incidence of *K. pneumoniae* in CAP cases, which is a significant concern and should be considered when treating CAP patients in India. The findings emphasize the importance of comprehensive diagnostic testing, including advanced methods such as bronchoalveolar lavage, urinary antigen tests, serology for atypical pathogens, and enzyme-linked immunosorbent assays, to improve diagnostic yield and guide targeted antibiotic therapy. This review underscores the need for updated empirical treatment guidelines that account for dominant pathogens. Future research should focus on employing advanced diagnostic methods to enhance understanding of CAP etiology.

## Introduction and background

Community-acquired pneumonia (CAP) is a significant worldwide health concern characterized by acute infections of the pulmonary parenchyma acquired outside hospital settings in individuals without recent hospitalizations. CAP is defined by new pulmonary infiltrates visible on chest radiographs or computed tomography scans, along with symptoms such as fever, cough, and dyspnea [1,2].

India is responsible for 23% of the global burden of CAP and 36% of the regional burden, as per the World Health Organization. It is estimated that approximately four million CAP cases occur annually in India, with 20% requiring hospitalization. The annual incidence rate of CAP in India is between five and 11 per 1,000 people. The mortality rate for CAP is under 5% among outpatients, about 10% for hospitalized patients, and can surpass 30% for those in intensive care units [3]. This underscores the urgent need for effective management strategies tailored to the Indian context.

Despite the significant burden of CAP in India, comprehensive data on its etiology in adult populations remains scarce. Previous studies have identified various pathogens as common causes of CAP, including *Streptococcus pneumoniae*, *Haemophilus influenzae*, *Staphylococcus aureus*, and atypical pathogens like *Mycoplasma pneumoniae *and *Legionella pneumophila *[4]. While previous studies have been conducted on pediatric populations, adolescents, and adults, there has been no recent systematic review specifically highlighting the adult population in India.

Given the variation in pathogen prevalence and diagnostic approaches, this systematic review aims to consolidate recent findings on the etiology of CAP among Indian adults. By synthesizing data from multiple studies focused on Indian adults, we seek to identify the most common causative agents of CAP and aid physicians in treating CAP patients across different regions of India.

## Review

Methods

**Figure 1:**
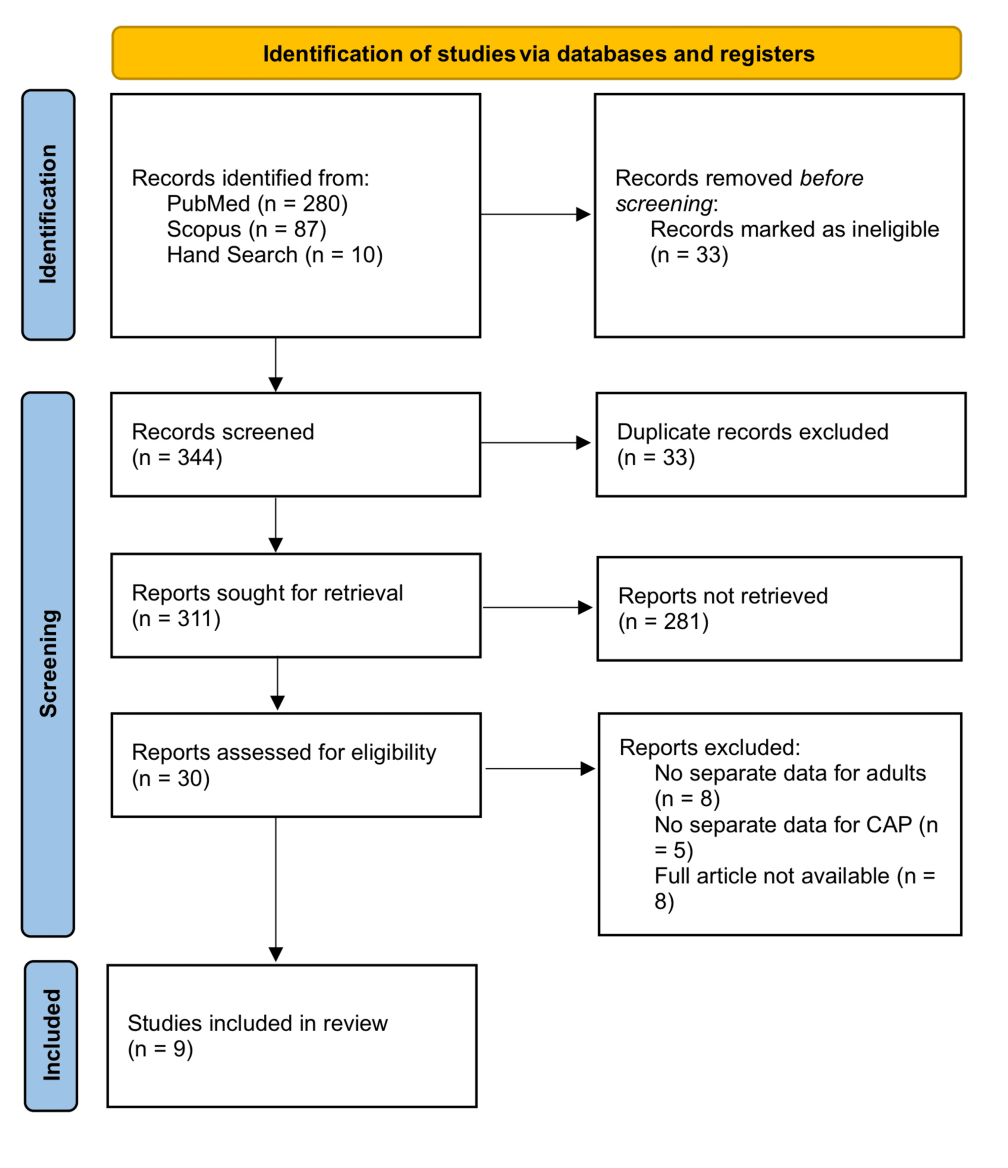
PRISMA flowchart of selected articles CAP, community-acquired pneumonia; PRISMA, Preferred Reporting Items for Systematic Reviews and Meta-Analyses

For this systematic review, we adhered to the Preferred Reporting Items for Systematic Reviews and Meta-Analyses (PRISMA) Guidelines 2020 [5]. The article selection process is comprehensively illustrated in the PRISMA flowchart (Figure 1).

Search Strategy

For our systematic review, we utilized the databases PubMed and Scopus-Elsevier and conducted hand searches of reference lists. Our search strategy was developed using key terms such as “Community-Acquired Pneumonia,” “CAP,” “INDIAN,” and “Adults.” Our database search was completed on April 14, 2024.

Inclusion Criteria

All studies, published between January 2010 and January 2024, that evaluated Indian adult patients with a confirmed diagnosis of CAP were eligible for inclusion. We decided to include only articles published in English. The types of studies included were primarily cohort studies, ensuring a robust and comprehensive dataset.

Exclusion Criteria

Studies were excluded if they involved CAP patients outside of India or were not conducted on the Indian population. Additionally, studies involving pediatric patients, articles published before 2010, or those written in languages other than English were excluded from the analysis. We also excluded case reports and case series to maintain the rigor and reliability of the dataset.

Data Management

A total of 344 results were retrieved by our search strategy. After removing 33 duplicates, we screened the titles and abstracts of the remaining 311 articles. This initial screening was performed individually by the first two authors, and their results were tallied. Based on our inclusion and exclusion criteria, 281 articles were excluded for the following reasons: 87 articles involved non-Indian patients, 53 articles included pediatric populations, 61 articles were published before 2010, five articles were not in English, 39 articles did not involve CAP patients, 28 were case reports, and eight were case series. The remaining 30 articles were read in full by all five authors, who collaboratively selected the final nine papers included in the systematic review.

Results

Search Results

Initially, we identified 280 articles through PubMed, 87 articles through Scopus, and 10 articles through hand searches, totaling 344 articles. After removing 33 duplicate reports, we screened 311 articles by their titles and abstracts. Based on our inclusion and exclusion criteria, 281 articles were excluded for the following reasons: 87 articles involved non-Indian patients, 61 articles were published before 2010, 53 articles included pediatric populations, 39 articles did not involve CAP patients, 28 were case reports, eight were case series, and five articles were not in English. We then reviewed the full text of 30 studies. Eight studies were excluded for not providing separate data for adults, five for not providing separate data for CAP, and another eight because the full article was not available. Finally, we included nine studies in our systematic review.

Study Characteristics

Table 1 provides a detailed description of our selected studies. The studies included a total of 1,643 adult inpatients diagnosed with CAP. All the studies were conducted in India.

**Table 1:** Overview of selected studies on CAP, highlighting the etiology and diagnostic tests used BAL: broncho-alveolar lavage; BC: blood culture; CAP: community-acquired pneumonia; CST: cross-sectional study; ELISA: enzyme-linked immunosorbent assay; IMF: immunofluorescence for IgM antibody against atypical bacteria and viruses (pneumoslide-M assay); OST, observational study; POS: prospective observational study; SC: sputum gram stain and culture; SMC: serology for Mycoplasma pneumoniae IgM and Chlamydophila pneumoniae IgM antibodies; UA: urinary antigen; VPCR: viral PCR

Study	Year published	Total patients	Study design	Diagnostic tests	Results
Dagaonkar et al. [6]	2012	100	POS	SC, BC, UA, and SMC	*Streptococcus pneumoniae* (23),* Klebsiella pneumoniae* (3),* Staphylococcus aureus* (1), *Mycoplasma pneumoniae* (5), *Legionella pneumophila* (3), *Chlamydia pneumoniae* (11), *Haemophilus influenzae* (9), *Pseudomonas aeruginosa* (2), *Moraxella catarrhalis* (6), Polymicrobial (6), and *Salmonella *Typhi (1)
Para et al. [7]	2018	225	POS	SC, BC, UA, and VPCR	*Streptococcus pneumoniae* (61), *Klebsiella pneumoniae* (11), *Staphylococcus aureus* (12), *Mycoplasma pneumoniae* (13), *Legionella pneumophila* (33), *Chlamydia pneumoniae* (10), *Pseudomonas* *aeruginosa* (7), *Moraxella* *catarrhalis* (9), Polymicrobial (9),* Influenza* A/B (13), *Mycobacterium tuberculosis* (11), *Acinetobacter baumannii *(2), and *Salmonella* Typhi (1)
Nagesh Kumar et al. [8]	2017	122	POS	SC, BC, and IMF	*Streptococcus pneumoniae* (19), *Klebsiella pneumoniae* (10), *Staphylococcus aureus* (4), *Mycoplasma pneumoniae* (9), *Legionella pneumophila* (7), *Haemophilus influenzae* (8), and *Pseudomonas* *aeruginosa* (4)
Mythri and Nataraju [9]	2013	100	CST	SC and BC	*Streptococcus pneumoniae *(10), *Klebsiella pneumoniae* (21), *Staphylococcus aureus* (4), *Mycoplasma pneumoniae* (9), *Legionella pneumophila *(7), and *Pseudomonas* *aeruginosa *(4)
Dorairaj et al. [10]	2015	107	CST	SC, BC, UA, SMC, and BAL	*Mycoplasma pneumoniae* (7) and *Chlamydia pneumoniae* (6)
Ravindranath and Raju [11]	2016	150	OST	SC and BC	*Streptococcus pneumoniae* (5), *Klebsiella pneumoniae* (5), *Staphylococcus aureus* (15), and *Pseudomonas* *aeruginosa* (1)
Jain et al. [12]	2014	120	POS	SC and BC	*Streptococcus pneumoniae* (20), *Klebsiella pneumoniae* (16), *Staphylococcus aureus* (11), *Haemophilus influenzae* (3), *Pseudomonas aeruginosa* (1), *Escherichia coli *(1), *Acinetobacter baumannii *(1), *Salmonella* Typhi (1), Enterobacter (1), and Citrobacter (1)
Menon et al. [13]	2013	145	POS	SC	*Streptococcus pneumoniae* (47), *Klebsiella pneumoniae* (29), *Pseudomonas* *aeruginosa* (13), and *Escherichia coli *(9)
Kalita et al. [14]	2021	574	POS	SC, BC, UA, SMC, BAL, and ELISA	*Streptococcus pneumoniae* (100), *Klebsiella pneumoniae* (102), *Staphylococcus aureus* (38), *Mycoplasma pneumoniae* (24), *Legionella pneumophila* (15), *Chlamydia pneumoniae* (7), *Haemophilus influenzae* (14), *Moraxella* *catarrhalis* (14), *Acinetobacter baumannii *(2), and Staphylococcus group (non-aureus) (1)

*Pathogen* *Distribution*

**Figure 2:**
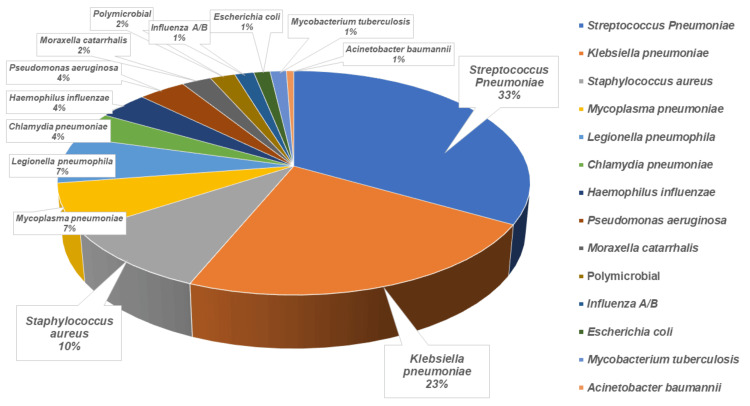
Results of the systematic review Displaying percentages of isolates

Analysis of the pooled data revealed that *S. pneumoniae *was the most common organism, accounting for 33% of the cases. This was followed by *Klebsiella pneumoniae *at 23%, *S. aureus *at 10%, *M. pneumoniae *and *L. pneumophila *each at 7%, and *Chlamydia pneumoniae*, *H. influenzae*, and *Pseudomonas aeruginosa*, each at 4%. Other pathogens were present in smaller quantities. The distribution of these pathogens, along with their respective percentages, is illustrated in a pie chart (Figure 2).

Antibiotic Sensitivity Distribution

**Figure 3:**
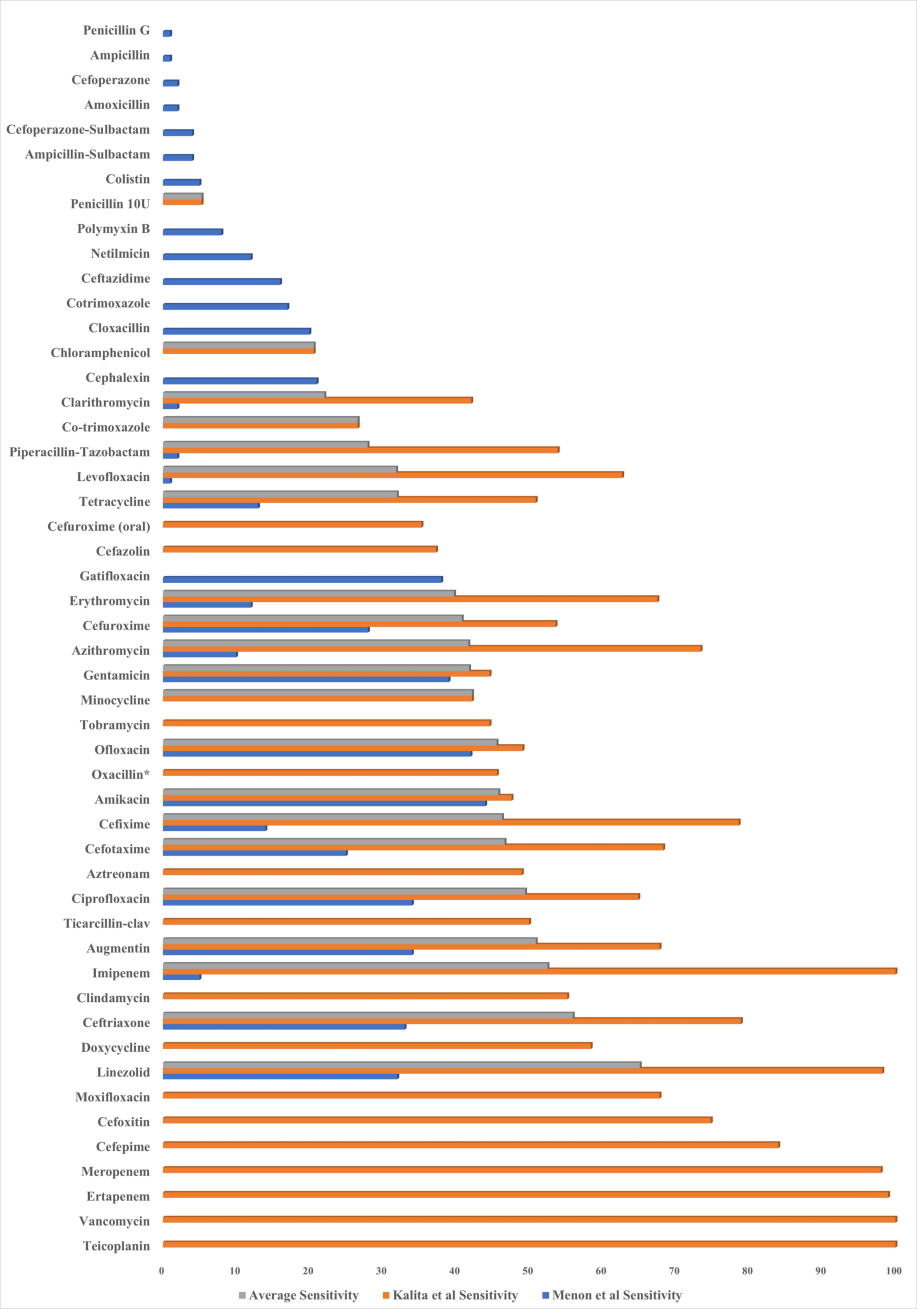
Antibiotic sensitivity pattern The chart presents a comparative analysis of antibiotic sensitivity among different studies, illustrating the sensitivity percentages of various antibiotics across three different datasets: average sensitivity (grey bars), Menon et al. [13] sensitivity (blue bars), and Kalita et al. [14] sensitivity (orange bars).

Only two studies, Menon et al. [13] and Kalita et al. [14], provided detailed antibiotic sensitivity data for CAP pathogens. The analysis based on pooled data from 719 patients from these studies illustrated in Figure 3 reveals significant variations in antibiotic sensitivity, underscoring the need for tailored empirical treatment based on local sensitivity patterns.

High sensitivity (80-100%) was observed for antibiotics such as vancomycin, teicoplanin, meropenem, ertapenem, and cefepime across all datasets. Additionally, clindamycin, linezolid, and doxycycline exhibited high sensitivity in the Kalita et al. study and in the average sensitivity data, consistently demonstrating strong effectiveness against the tested pathogens and suggesting their potential as reliable treatment options.

Moderate sensitivity (50-79%) was noted for antibiotics including ciprofloxacin, ceftriaxone, augmentin, and imipenem in both the Kalita et al. and average sensitivity data. Levofloxacin and tetracycline showed moderate sensitivity in the Menon et al. study and the average sensitivity data. These antibiotics may still be effective but could face varying resistance levels depending on the local microbial landscape.

Low sensitivity (<50%) was observed for antibiotics such as penicillin G, ampicillin, cefoperazone, amoxicillin, cefoperazone-sulbactam, and ampicillin-sulbactam across all datasets. Colistin, polymyxin B, netilmicin, and ceftazidime also exhibited low sensitivity in the Kalita et al. study and average sensitivity data, indicating limited effectiveness and higher resistance levels for these antibiotics.

Confusion, Urea, Respiratory Rate, Blood Pressure, Age ≥65 Years (CURB-65) Score

In the study by Ravindranath and Raju, the CURB-65 scoring system was used to evaluate the severity of CAP in patients. The findings demonstrated a clear correlation between higher CURB-65 scores and increased mortality and ICU admission rates. For patients with scores of 0 (Class 0) and 1 (Class I), there were no deaths or ICU admissions among the 27 (18%) and 31 (20.7%) patients, respectively. In patients with a score of 2 (Class II), none of the 42 (28%) patients died, although five (11.9%) required ICU admission. Among those with a score of 3 (Class III), two (9.52%) out of 21 (14%) patients died, and nine (42.86%) needed ICU care. The mortality rate increased significantly to 11 (47.82%) for those with a score of 4 (Class IV), with 20 (86.96%) out of 23 (15.3%) requiring ICU admission. In the highest severity group, with a score of 5 (Class V), three (50%) out of six (4.0%) patients died, and all six required ICU care. Furthermore, a CURB-65 score greater than 2 was identified as an independent risk factor for mortality. The sensitivity and specificity for CURB-65 risk class >III to predict death were 100% and 74.62%, respectively, with a positive predictive value of 32% and a negative predictive value of 100% [11].

Additionally, infections caused by typical pathogens were associated with higher CURB-65 scores at admission, more bilateral radiographic involvement, and worse clinical outcomes such as septic shock and the need for mechanical ventilation. In contrast, infections by atypical pathogens generally resulted in milder cases [7].

Discussion

Regional Variation in the Predominant Pathogen

In this review, *S. pneumoniae* was identified as the most common organism, found in approximately 33% of cases across the studies, which aligns with its global recognition as a leading cause of CAP [2,15]. For instance, Dagaonkar et al. in Mumbai, Para et al. in Kashmir, and Nagesh Kumar et al. identified *S. pneumoniae *as the predominant organism in their respective regions [6-8].

Conversely, some studies highlighted different leading pathogens. For example, Mythri and Nataraju in Bengaluru reported* K. pneumoniae *as the most common pathogen [9]. This pattern was also observed in the study by Kalita et al., indicating a significant presence of *K. pneumoniae *in community-acquired cases [14], which is noteworthy as it is not typically a common pathogen in CAP cases. Additionally, Ravindranath and Raju in Telangana found *S. aureus *to be the most prevalent pathogen [11].

*H. influenzae*, recognized globally as a common cause of CAP, was found in only about 4% of cases in this review. This discrepancy underscores the need for region-specific etiological studies to accurately understand and address the causes of pneumonia in different populations [2,15,16].

Diagnostic Yield

The study by Dorairaj et al. in Chennai and Jain et al. from Madhya Pradesh noted a lower diagnostic yield due to the limited use of advanced diagnostic tools, primarily relying on sputum and blood cultures [10,12]. This limitation was echoed by Ravindranath and Raju, who reported a low etiological yield, cautioning against definitive conclusions due to this limitation [11]. Mythri and Nataraju attributed their low isolation rate to factors such as the nonavailability of sputum samples and prior antibiotic use [9]. The need for comprehensive diagnostic testing is further emphasized by the findings of Kalita et al., who utilized a range of diagnostic methods, including urinary antigen tests for *Legionella *and *Streptococcus *serology for atypical pathogens, enzyme-linked immunosorbent assay (ELISA), and bronchoalveolar lavage, resulting in the highest diagnostic yield [14].

Antibiotic Sensitivity and Resistance

The comparative analysis of antibiotic sensitivity data from Kalita et al. and Menon et al. reveals significant variations in the effectiveness of antibiotics against CAP pathogens. Kalita et al. reported a higher sensitivity for a broader range of antibiotics, while Menon et al. observed generally lower sensitivity percentages. This underscores the necessity for ongoing surveillance of antibiotic sensitivity to ensure treatment guidelines are up to date and effective. Tailoring empirical treatment based on local data is crucial for optimizing patient outcomes and managing antibiotic resistance effectively [13,14].

A significant concern highlighted by the studies is the high resistance rates among key pathogens. In Kalita et al., *K. pneumoniae* isolates showed high resistance to penicillin/beta-lactamase inhibitors and cephalosporins, although carbapenems remained effective. *S. pneumoniae* strains also exhibited substantial multidrug resistance. Menon et al. noted low sensitivity rates to macrolides among common pathogens, emphasizing the need for revised antibiotic strategies. These findings underscore the critical importance of incorporating local antibiotic sensitivity data into treatment protocols to effectively combat resistance and improve patient outcomes in CAP management [13,14].

CURB-65 Score

It is a valuable tool for predicting outcomes for patients with CAP. As the CURB-65 score increases, there is a corresponding rise in both mortality and the need for ICU admission, emphasizing the importance of this scoring system in clinical settings. Specifically, patients with scores of 0 and 1 had favorable outcomes with no deaths or ICU admissions. However, the severity of the condition and adverse outcomes, such as death and ICU admission rates, significantly increased with higher CURB-65 scores [7,11].

The study also highlighted that infections caused by typical pathogens were linked to higher CURB-65 scores at admission, which were associated with more severe cases, including greater bilateral radiographic involvement and worse clinical outcomes such as septic shock and the need for mechanical ventilation. Conversely, infections by atypical pathogens typically result in milder cases. These findings underscore the necessity of considering CURB-65 scores in the management and treatment plans for patients with CAP, as scores greater than 2 are significant predictors of mortality. The sensitivity and specificity metrics further validate the CURB-65 score’s effectiveness in predicting patient outcomes, reinforcing its role as an essential tool in clinical practice [7,11].

Comparison with Reviews Outside India

A systematic review conducted in Ethiopia by Seid et al., which included 2,496 participants, found *K. pneumoniae* to be the most common pathogen, followed by *S. pneumoniae *and *S. aureus *[17]. In contrast, a study conducted by the British Thoracic Society involving 453 adults across 25 British hospitals identified *S. pneumoniae *as the predominant pathogen, followed by *M. pneumoniae *and *Influenza A *virus [18]. These variations underscore the significant regional differences in the etiology of CAP, highlighting the importance of continuously updating regional data on CAP pathogens.

The key findings of this review are presented in Table 2.

**Table 2:** Key points of the review CAP, community-acquired pneumonia; CURB-65, Confusion, Urea, Respiratory rate, Blood pressure, age ≥65 years

Key points
*Streptococcus pneumoniae* was identified as the most common pathogen in CAP among Indian adults, followed by *Klebsiella pneumoniae* and* Staphylococcus aureus*. Physicians in India should ensure their coverage of empirical therapy for CAP patients.
The diagnostic yield varied significantly across studies, with higher yields associated with advanced diagnostic tools such as urinary antigen tests and bronchoalveolar lavage. Comprehensive diagnostic testing, including these advanced methods, is needed to improve diagnostic yield and guide targeted antibiotic therapy.
High sensitivity was observed for antibiotics such as vancomycin, teicoplanin, meropenem, ertapenem, and cefepime. Low sensitivity was observed for penicillin G, ampicillin, cefoperazone, amoxicillin, cefoperazone-sulbactam, ampicillin-sulbactam, and ceftazidime, indicating limited effectiveness and higher resistance levels.
High resistance rates among key pathogens like *Klebsiella pneumoniae* and *Streptococcus pneumoniae* underscore the importance of incorporating local antibiotic sensitivity data into treatment protocols.
The CURB-65 scoring system proved effective in predicting outcomes in CAP patients, with higher scores correlating with increased mortality and ICU admission rates, highlighting its importance in clinical settings.

Limitations

This systematic review concentrated on data from 2010 onward, which may also be a limitation as it excluded studies published earlier. Additionally, our review exclusively included articles published in English, potentially leading to the exclusion of relevant research published in other languages. Furthermore, none of the final studies included in our review contained outpatient data, which limits the generalizability of the findings to hospitalized patients only. Most studies relied primarily on sputum cultures, which often had a low diagnostic yield. Moreover, testing for viral causes of pneumonia was not conducted in the majority of studies. These limitations highlight the need for more studies utilizing comprehensive diagnostic tests, such as bronchoalveolar lavage, urine antigen tests, serology for atypical pathogens, and ELISA, to gain a better understanding of CAP etiology in India.

## Conclusions

This systematic review consolidates key findings on the etiology of CAP among Indian adults, identifying *S. pneumoniae*, *K. pneumoniae*, and *S. aureus *as the predominant pathogens. *S. pneumoniae *remains the most common causative agent, aligning with global trends, while the rising incidence of* K. pneumoniae *warrants particular attention in the Indian context. The review underscores the necessity for advanced diagnostic approaches to improve pathogen identification and guide targeted antibiotic therapy. Integrating techniques such as bronchoalveolar lavage, urinary antigen tests, serology for atypical pathogens, and ELISA is crucial to enhance diagnostic accuracy.

Antibiotic resistance patterns observed highlight significant regional variations, emphasizing the need for continuous local surveillance to inform empirical treatment guidelines. High resistance rates among key pathogens underscore the importance of incorporating local antibiotic sensitivity data into treatment protocols to optimize patient outcomes and manage antibiotic resistance effectively. Future research should focus on employing advanced diagnostic techniques and maintaining updated regional data on CAP pathogens to ensure empirical treatments remain effective and relevant. Continuous efforts are needed to improve diagnostic yield and address the challenges posed by antibiotic resistance, ultimately enhancing the management of CAP in Indian adults.
