# Correction: A Systematic Review of Community-Acquired Pneumonia in Indian Adults

**DOI:** 10.7759/cureus.c265

**Published:** 2025-08-26

**Authors:** Vikram B Vikhe, Ahsan A Faruqi, Rahul S Patil, Avani Reddy, Devansh Khandol

**Affiliations:** 1 General Medicine, Dr. D. Y. Patil Medical College, Hospital & Research Centre, Dr. D. Y. Patil Vidyapeeth, Pune (Deemed to be University), Pune, IND

This article has been corrected as follows:

- The PRISMA diagram has been updated to indicate that 33 records were marked as ineligible and removed before screening.
- "Displaying percentages of isolates" has been added to the Figure 2 legend.

